# Supplementary material for: Evidence of renal angiomyolipoma neoplastic stem cells arising from renal epithelial cells
Source: Nat Commun. 2017 Nov 13;8:1466. doi: 10.1038/s41467-017-01514-3 (PMC5684212; doi:10.1038/s41467-017-01514-3)
Supplement: Supplementary file 1 — Supplementary Information [file 41467_2017_1514_MOESM1_ESM.pdf]

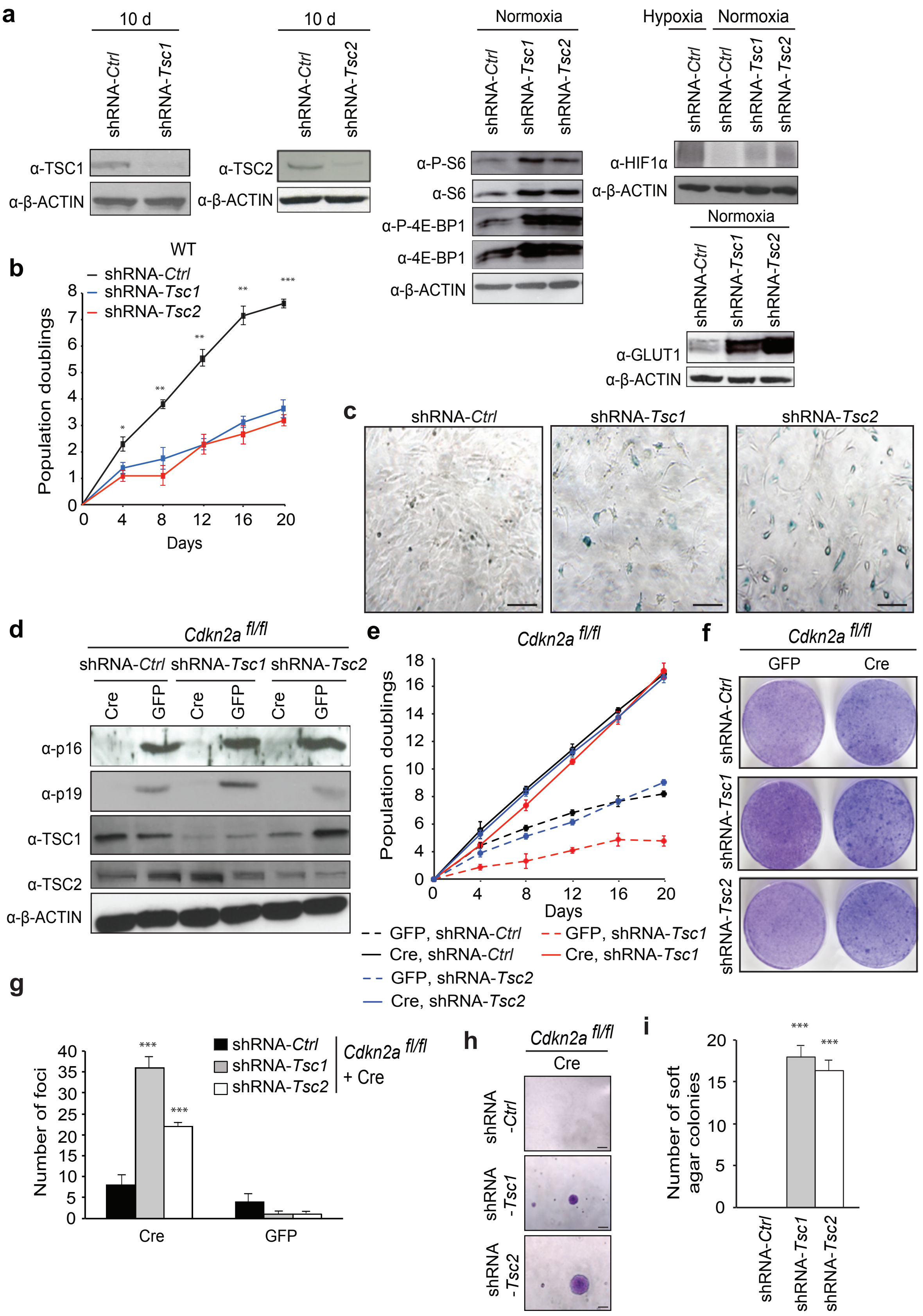

Supplementary Figure 1. Combined loss-of-function of *Tsc1* or *Tsc2* with *Cdkn2a* transforms primary MEFs

(a) Western blot analysis for the indicated proteins in wild type MEFs infected with lentiviruses expressing an empty shRNA (shRNA-*Ctrl*), an shRNA targeting *Tsc1* (shRNA-*Tsc1*) or *Tsc2* (shRNA-*Tsc2*) grown under normoxia (5% O_2) or hypoxia (1% O_2) as indicated. P-S6 = phosphorylated Ser240/244 ribosomal protein S6 (P-S6) and P-4E-BP1 = phosphorylated Thr37/46 4E-BP1. **(b)** Proliferation assay of wild type MEFs infected with lentiviruses expressing an empty shRNA (shRNA-*Ctrl*), an shRNA targeting *Tsc1* (shRNA-*Tsc1*) or *Tsc2* (shRNA-*Tsc2*). **(c)** Senescence associated β-Galactosidase staining of wild type MEFs 8 days after infection with the indicated lentiviral knockdown vectors. Scale bars: 50 μm. **(d-i)** *Cdkn2a*$^{fl/fl}$ MEFs were infected with adenovirus expressing GFP or Cre and lentiviruses expressing an empty shRNA (shRNA-*Ctrl*), an shRNA targeting *Tsc1* (shRNA-*Tsc1*) or *Tsc2* (shRNA-*Tsc2*). **(d)** Western blotting confirmed the anticipated genetic changes. Cellular transformation was assessed by **(e)** proliferation assay, **(f,g)** ability of genetically altered cells to form foci (shown by crystal violet staining in (f) and quantified in (g)) 14 days after plating 1 x 10^4 test cells with 5 x 10^5 wild type cells and **(h,i)** ability of genetically altered cells to form colonies in soft agar (shown by shown by crystal violet staining in (h) and quantified in (i)) 14 days after plating 2.5 x 10^4 cells/well. Scale bars: 200 μm. All graphs depict mean ± SD. Student's *t* test, n=3. ** P <0.01; *** P <0.001.

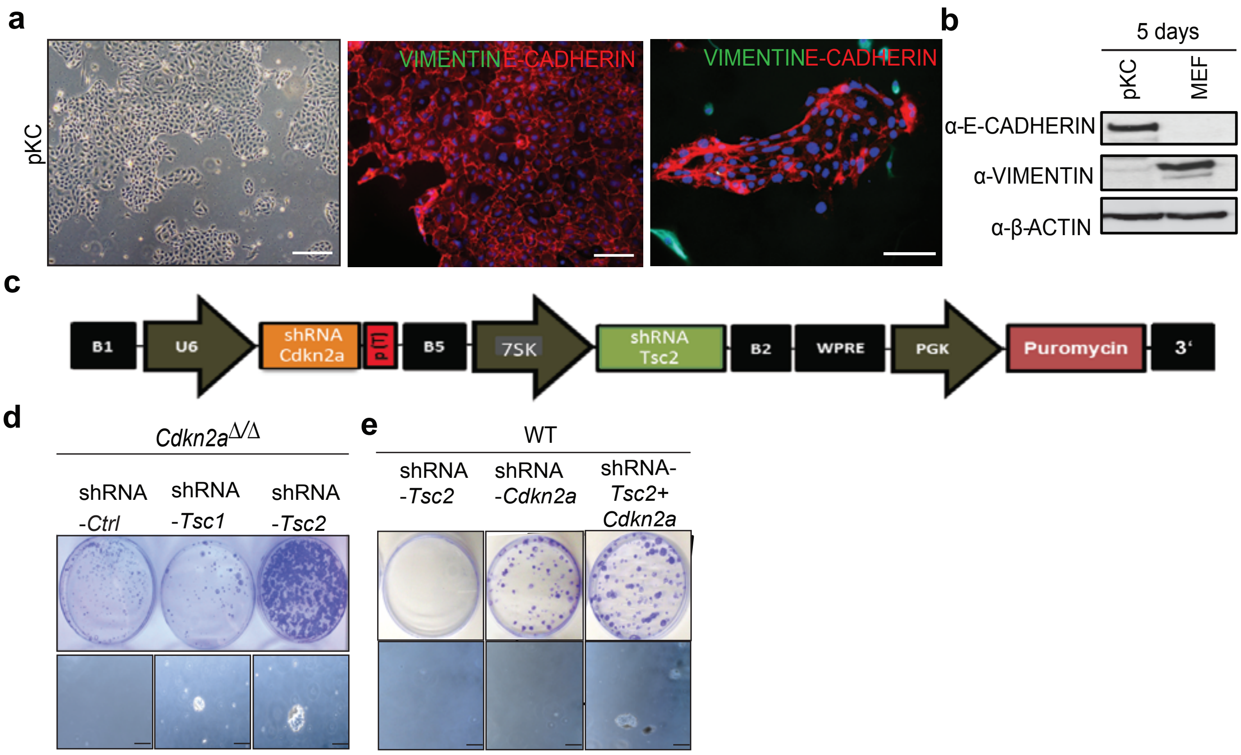

Supplementary Figure 2. Combined loss-of-function of *Tsc1* or *Tsc2* with *Cdkn2a* transforms primary kidney cells
(a) Phase contrast images of WT primary kidney epithelial cells and immunofluorescence staining of the same cells for VIMENTIN and E-CADHERIN. Scale bars: 50 µm. **(b)** Western blot analysis of cultures of primary kidney cells (pKC) and MEFs 5 days after cell seeding. **(c)** Schematic of the MuLE lentiviral vector that was generated to simultaneously knockdown *Cdkn2a* and *Tsc2.* **(d,e)** Crystal Violet staining of engineered cells of the indicated genotypes 14 days after seeding at low density (2 x 10^3 cells per plate) and representative images of the same cells seeded in a soft agar colony assay (2.5 x 10^4 cells/well) after 3 weeks of growth. Scale bars: 200 µm.

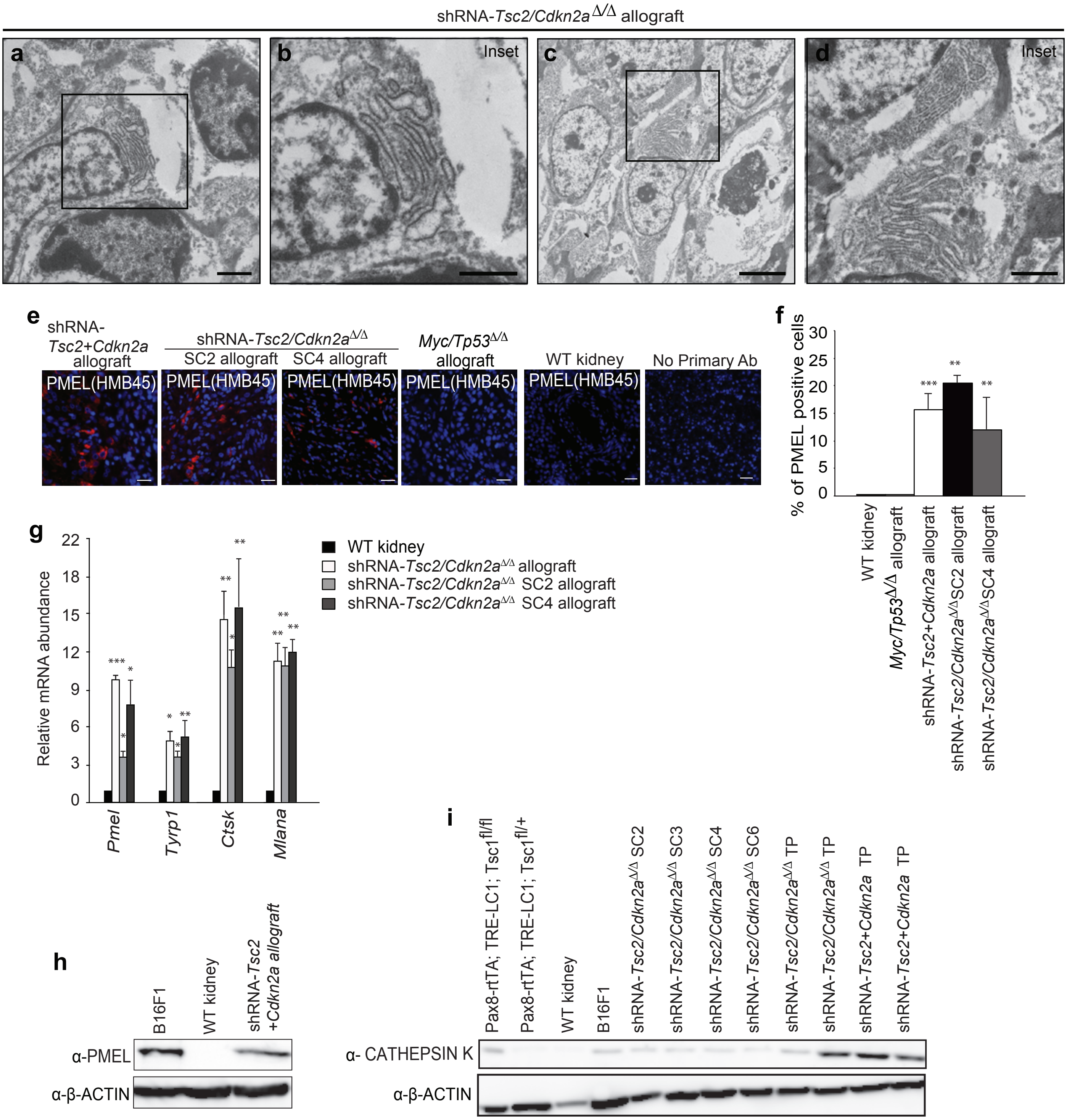

Supplementary Figure 3. Characterisation of AML-like allografts derived from *Tsc2/Cdkn2a* deficient spheres

(a-d) Electron microscopy revealing expanded endoplasmic reticulum in shRNA-*Tsc2/Cdkn2a*$^{\Delta/\Delta}$ AML-like allograft tumour cells. b and d represent zooms of the boxed regions in a and c respectively. Scale bars: 500 nm. **(e)** Representatitive immunofluorescence staining of PMEL(HMB45) in shRNA-*Tsc2+Cdkn2a* allograft, shRNA-*Tsc2/Cdkn2a*$^{\Delta/\Delta}$ allografts from sphere clones SC2 and SC4 and as negative controls a *Myc/Tp53*$^{\Delta/\Delta}$ allograft tumour, wild type normal kidney and shRNA-*Tsc2+Cdkn2a* allograft with no primary antibody. Scale bars: 50 µm. **(f)** Quantification of percentage of PMEL positive cells in the indicated samples. Graph depicts mean ± s.d. Student's *t* test, n=3. ** P <0.01; *** P <0.001. **(g)** Real time PCR analysis of the indicated genes in WT kidney, shRNA-*Tsc2/Cdkn2a*$^{\Delta/\Delta}$ allograft derived from total sphere population and from SC2 and SC4. Graph depicts mean ± s.d. Student's *t* test, n=3. ** P <0.01; *** P <0.001. **(h)** Western blot analysis of PMEL in WT kidney and shRNA-*Tsc2+Cdkn2a* allograft. B16F1 mouse melanoma cell line was used as a positive control for PMEL expression in this assay. **(i)** Western blot analysis of CATHEPSIN K in *Pax8-rtTA; TRE-LC1; Tsc1*$^{fl/fl}$, *Pax8-rtTA; TRE-LC1; Tsc1*$^{fl/+}$ and WT kidneys and in shRNA-*Tsc2/Cdkn2a*$^{\Delta/\Delta}$ allografts from sphere clones (SC) or total sphere populations (TP) or allografts from shRNA-*Tsc2+Cdkn2a* total sphere populations.

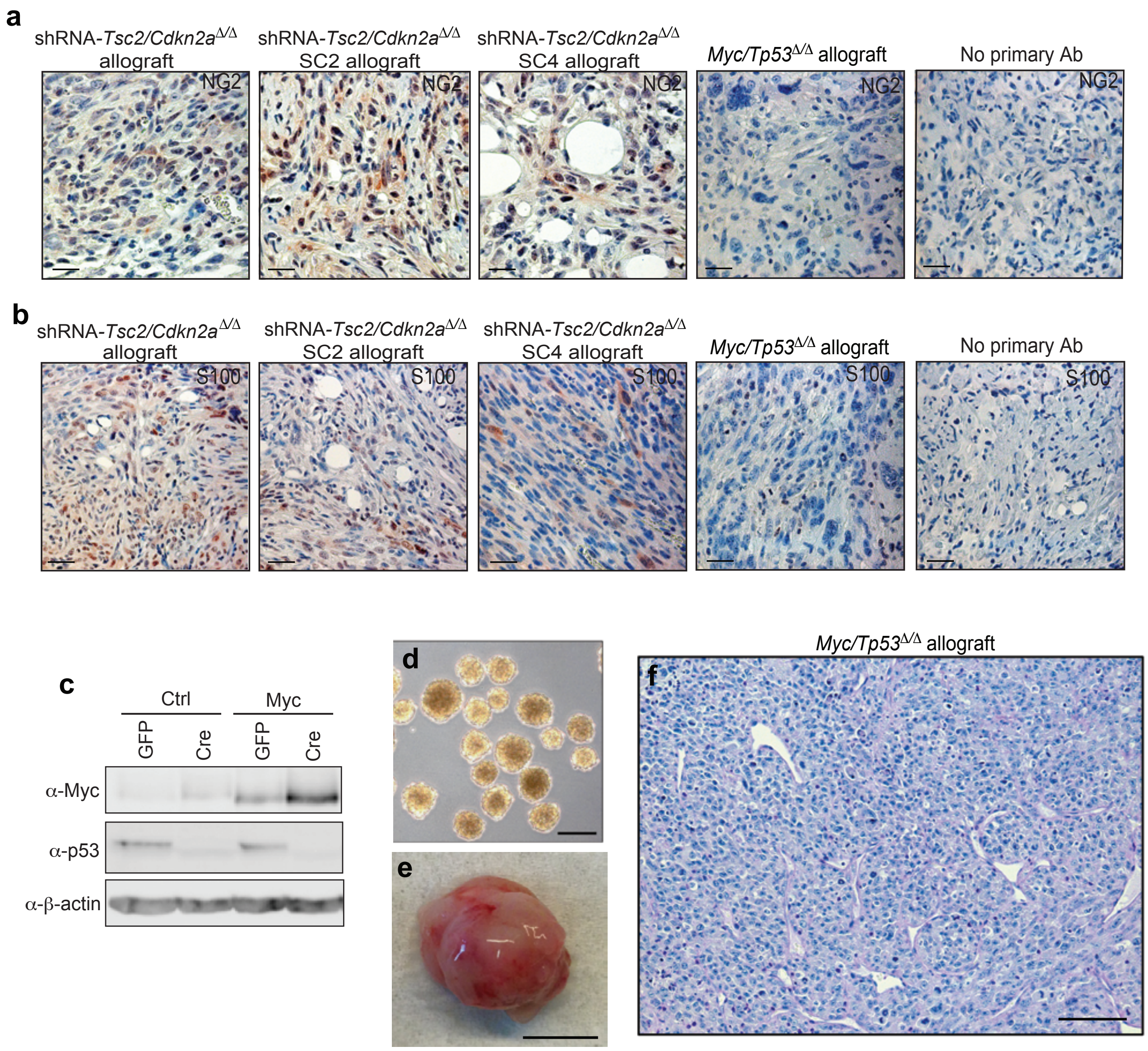

Supplementary Figure 4. Characterization of shRNA-*Tsc2/Cdkn2a*$^{\Delta/\Delta}$ allografts

(a,b) Representative NG2 and S100 immunohistochemical stainings in shRNA-*Tsc2/Cdkn2a*$^{\Delta/\Delta}$ allografts from total sphere population, SC2 and SC4, *Myc/Trp53*$^{\Delta/\Delta}$ allograft and shRNA-*Tsc2/Cdkn2a*$^{\Delta/\Delta}$ allograft without primary antibody. Scale bars: 50 µm. **(c)** Western blot of *Trp53*$^{fl/fl}$ primary kidney cells infected with adenovirus expressing GFP or Cre and lentiviruses expressing nothing (empty) or *Myc*. **(d)** Phase contrast images of spheres formed in suspension culture conditions by *Myc/Trp53*$^{\Delta/\Delta}$ primary kidney cells. Scale bar: 100 µm **(e)** *Myc/Trp53*$^{\Delta/\Delta}$ allograft derived from subcutaneous injections of *Myc/Trp53*$^{\Delta/\Delta}$ primary kidney cells into *SCID-beige* mice. Scale bar: 5 mm **(f)** Representative H&E staining of a *Myc/Trp53*$^{\Delta/\Delta}$ allograft tumour. Scale bars: 100 µm.

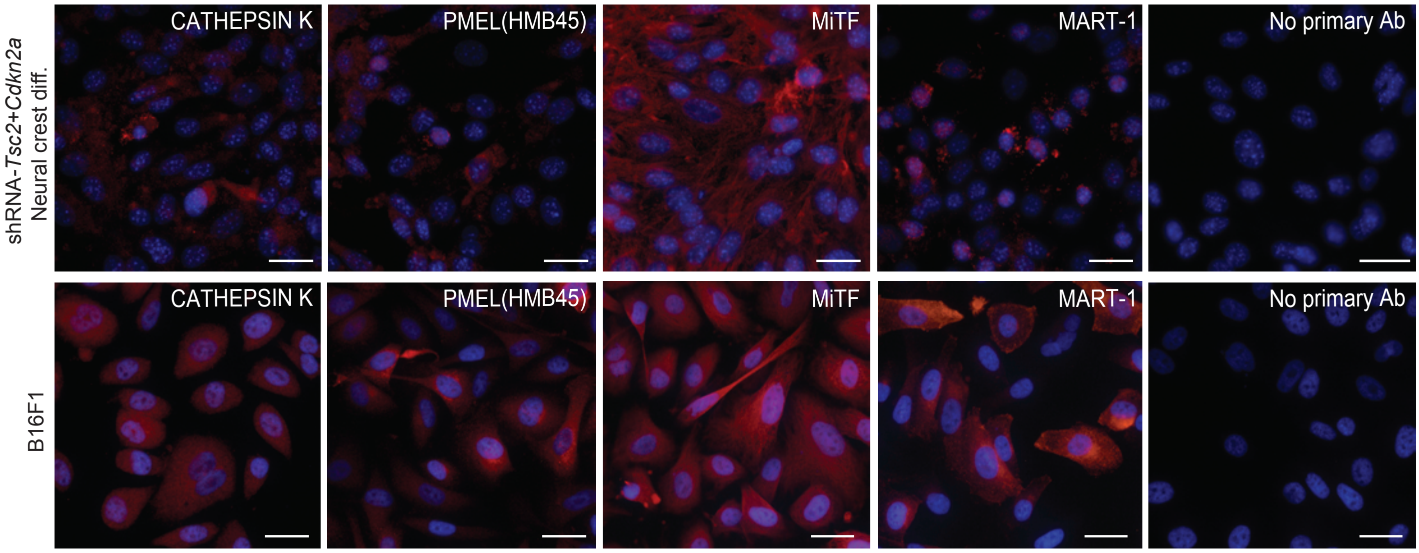

Supplementary Figure 5. Neural crest-differentiated shRNA-*Tsc2+Cdkn2a* spheres express melanocytic markers

(a) Immunofluorescence staining using antibodies against CATHEPSIN K, PMEL(HMB45), MiTF and MART-1 in adherent neural crest-differentiated shRNA-*Tsc2+Cdkn2a* sphere cells (upper panel) and in the mouse melanoma cell line B16F1 (lower panel). Scale bars: 50 µm.

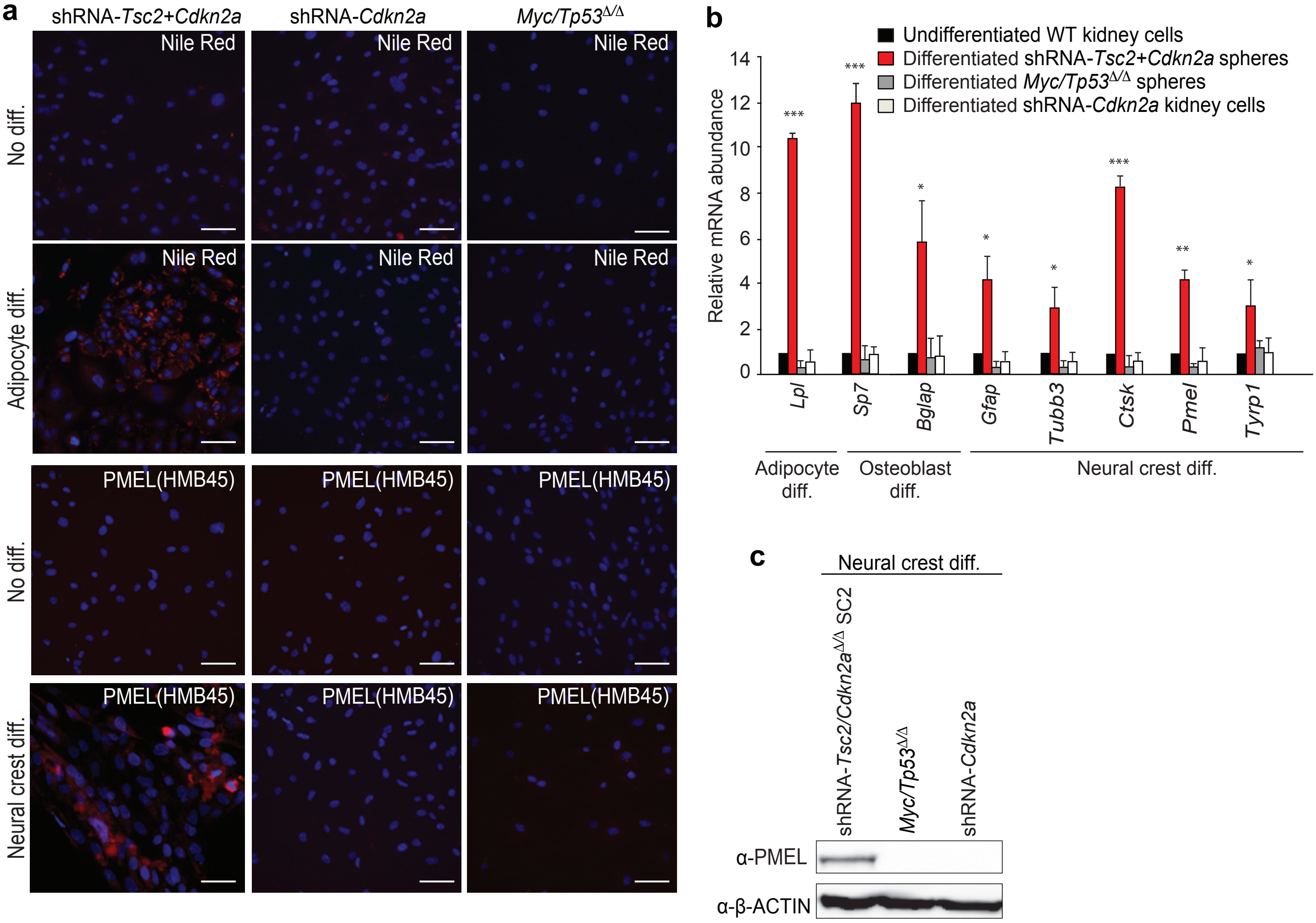

Supplementary Figure 6. Differentiation capacity of AML-like spheres is TSC2 dependent.
(a) Nile Red staining and PMEL(HMB45) immunofluorescence staining of shRNA-*Tsc2+Cdkn2a* sphere cells (derived from *Pax8-rtTA; TRE-LC1; R26R-tdTomato* mice), shRNA-*Cdkn2a* kidney cells and *Myc/Trp53*$^{\Delta/\Delta}$ sphere cells cultured in specific adipocyte- and neural crest-differentiation media. Scale bars: 50 µm. **(b)** mRNA expression analysis of the indicated genes in cultures of shRNA-*Tsc2+Cdkn2a* sphere cells (derived from *Pax8-rtTA; TRE-LC1; R26R-tdTomato* mice), shRNA-*Cdkn2a* kidney cells and *Myc/Trp53*$^{\Delta/\Delta}$ sphere cells that were differentiated in adipocyte-, osteoblast- and neural crest-differentiation media compared with undifferentiated WT primary kidney cells. Graph depicts mean ± s.d. Student's *t* test, n=3. ** P <0.01; *** P <0.001. **(c)** Western blot analysis of PMEL in neural crest-differentiated cells from shRNA-*Tsc2/Cdkn2a*$^{\Delta/\Delta}$ SC2 spheres, *Myc/Trp53*$^{\Delta/\Delta}$ spheres and from shRNA-*Cdkn2a*$^{\Delta/\Delta}$ cells.

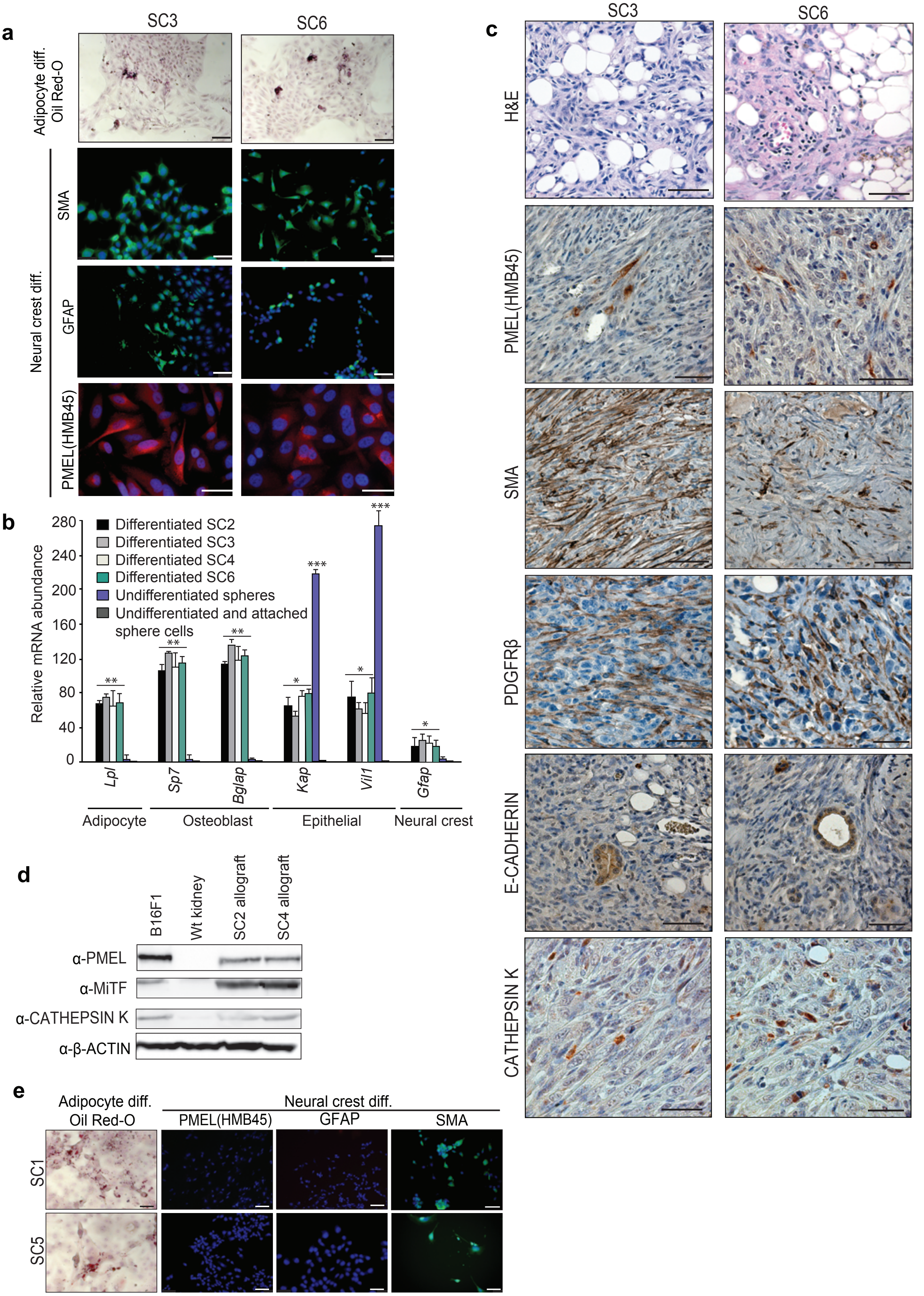

Supplementary Figure 7. Cells from *Tsc2/Cdkn2a* deficient spheres exhibit stem cell-like characteristics
(a) shRNA-*Tsc2/Cdkn2a*$^{\Delta/\Delta}$ SC3 and SC6 were cultured in specific adipocyte- and neural crest-differentiation media and stained immunofluorescently using antibodies against SMA, GFAP and PMEL(HMB45) or stained with Oil-Red-O. Scale bars: 50 µm. **(b)** mRNA expression analysis of indicated genes in shRNA-*Tsc2/Cdkn2a*$^{\Delta/\Delta}$ sphere clones SC2, SC3, SC4 and SC6 that were differentiated in adipocyte-, osteoblast-, epithelial and neural crest-differentiation media compared with undifferentiated shRNA-*Tsc2/Cdkn2a*$^{\Delta/\Delta}$ total population floating spheres and shRNA-*Tsc2/Cdkn2a*$^{\Delta/\Delta}$ total population sphere cells that attached in the absence of specific differentiation media (undifferentiated and attached sphere cells). Graph depicts mean ± s.d., n=3. Student's *t* test, n=3. * P <0.05; ** P <0.01; *** P <0.001. **(c)** Allograft tumours obtained from shRNA-*Tsc2/Cdkn2a*$^{\Delta/\Delta}$ SC3 and SC6. Immunohistochemical stainings of PMEL(HMB45), SMA, PDGFRβ, E-CADHERIN and CATHEPSIN K. Scale bars: 50 µm. **(d)** Western blot analysis of PMEL, MiTF and CATHEPSIN K in allografts from shRNA-*Tsc2/Cdkn2a*$^{\Delta/\Delta}$ SC2 and SC4 spheres. B16F1 mouse melanoma cell line and WT kidney lysates were used as positive and negative control respectively for melanocytic protein expression in this assay **(e)** Two additional shRNA-*Tsc2/Cdkn2a*$^{\Delta/\Delta}$ sphere clones (SC1 and SC5) not able to generate allografts were cultured in specific adipocyte- and neural crest-differentiation media and stained using antibodies against PMEL(HMB45), GFAP and SMA or stained with Oil-Red-O to evaluate their differentiation capacity. Scale bars: 50 µm.

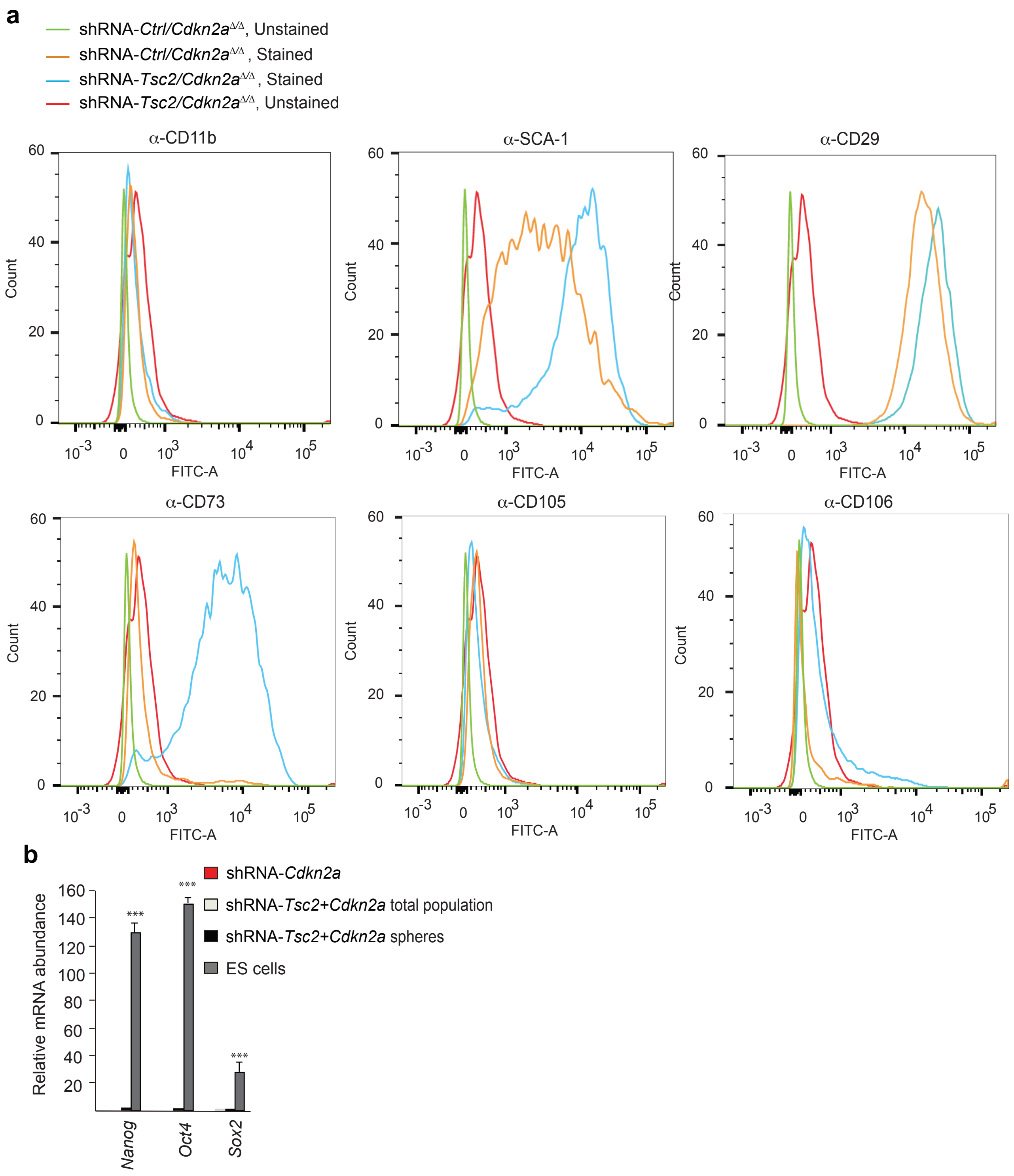

Supplementary Figure 8. AML-forming sphere cells differ molecularly from mesenchymal stem cells and ES cells
(a) Flow cytometry analyses of sphere-selected shRNA-*Tsc2*/*Cdkn2a*$^{\Delta/\Delta}$ cells and control *Cdkn2a*$^{\Delta/\Delta}$ kidney cells using antibodies against the mesenchymal stem cell markers CD29, Sca-1, CD105, CD106 and CD73 (including CD11b as a negative control). **(b)** Expression levels of embryonic stem cell markers (*Nanog*, *Oct4* and *Sox2)* in shRNA-*Tsc2+Cdkn2a* infected total population of kidney cells (total population), spheres, shRNA-*Cdkn2a* infected total population of kidney cells and ES cells. Graph depicts mean ± s.d. Student's *t* test, n=3. *** P <0.001.

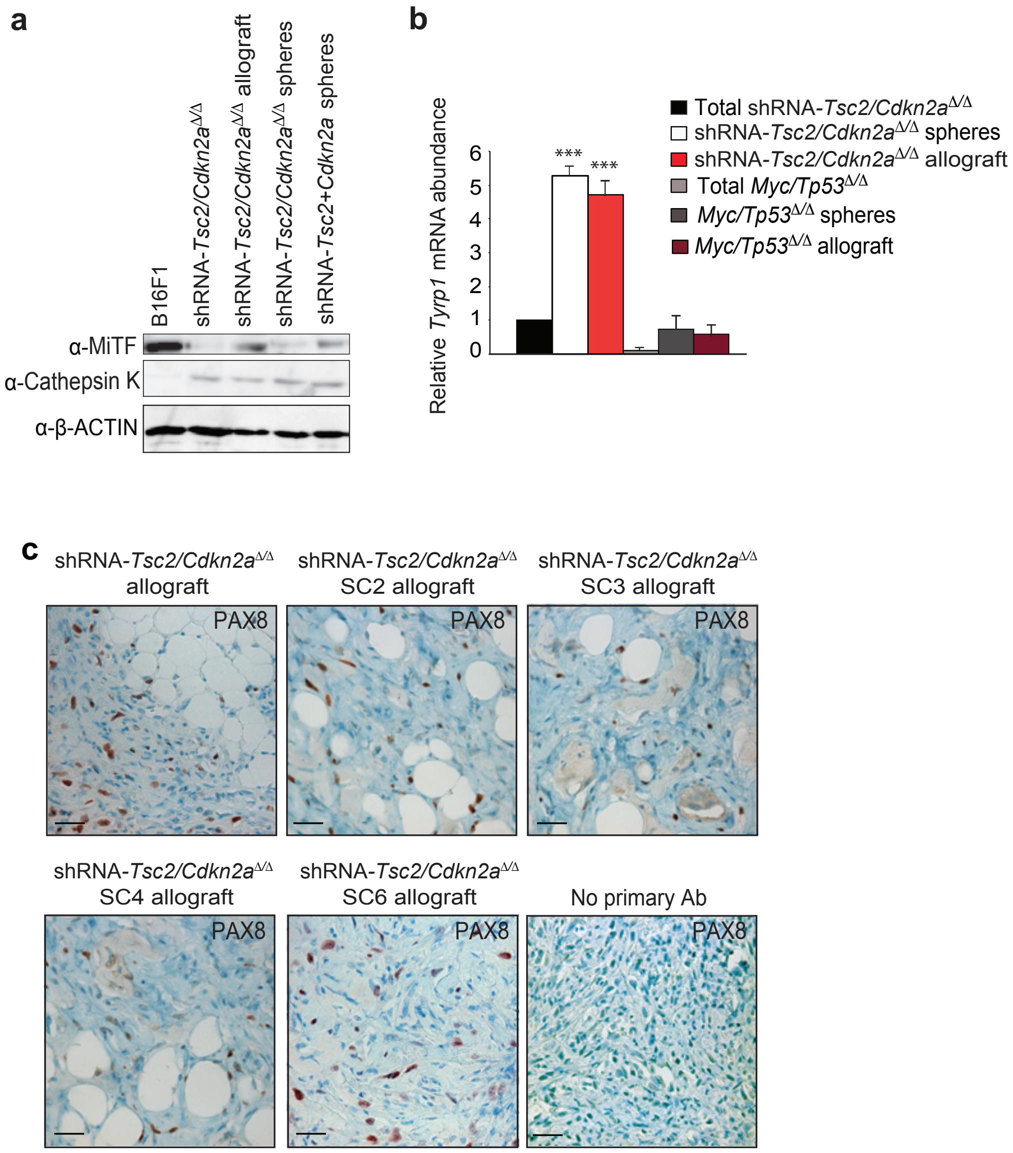

Supplementary Figure 9. Additional molecular analyses of AML-forming spheres and allografts
(a) Western blot analysis of MiTF and CATHEPSIN K in shRNA-*Tsc2/Cdkn2a*$^{\Delta/\Delta}$ spheres and allografts in comparison to shRNA-*Tsc2/Cdkn2a*$^{\Delta/\Delta}$ total population non-sphere selected kidney cultured cells and the B16F1 mouse melanoma cell line. **(b)** mRNA expression analysis of *Tyrp1* in shRNA-*Tsc2/Cdkn2a*$^{\Delta/\Delta}$ total population non-sphere selected kidney cultured cells, spheres and allografts and in *Myc/Trp53*$^{\Delta/\Delta}$ adherent kidney cells, spheres and allograft. Graph depicts mean ± SD. Student's *t* test, n=3. ** P <0.01; *** P <0.001. **(c)** PAX8 immunohistochemical stainings of shRNA-*Tsc2/Cdkn2a*$^{\Delta/\Delta}$ allografts from total sphere population and from sphere clones (SC2, SC3, SC4, SC6) Scale bars: 50 μm.

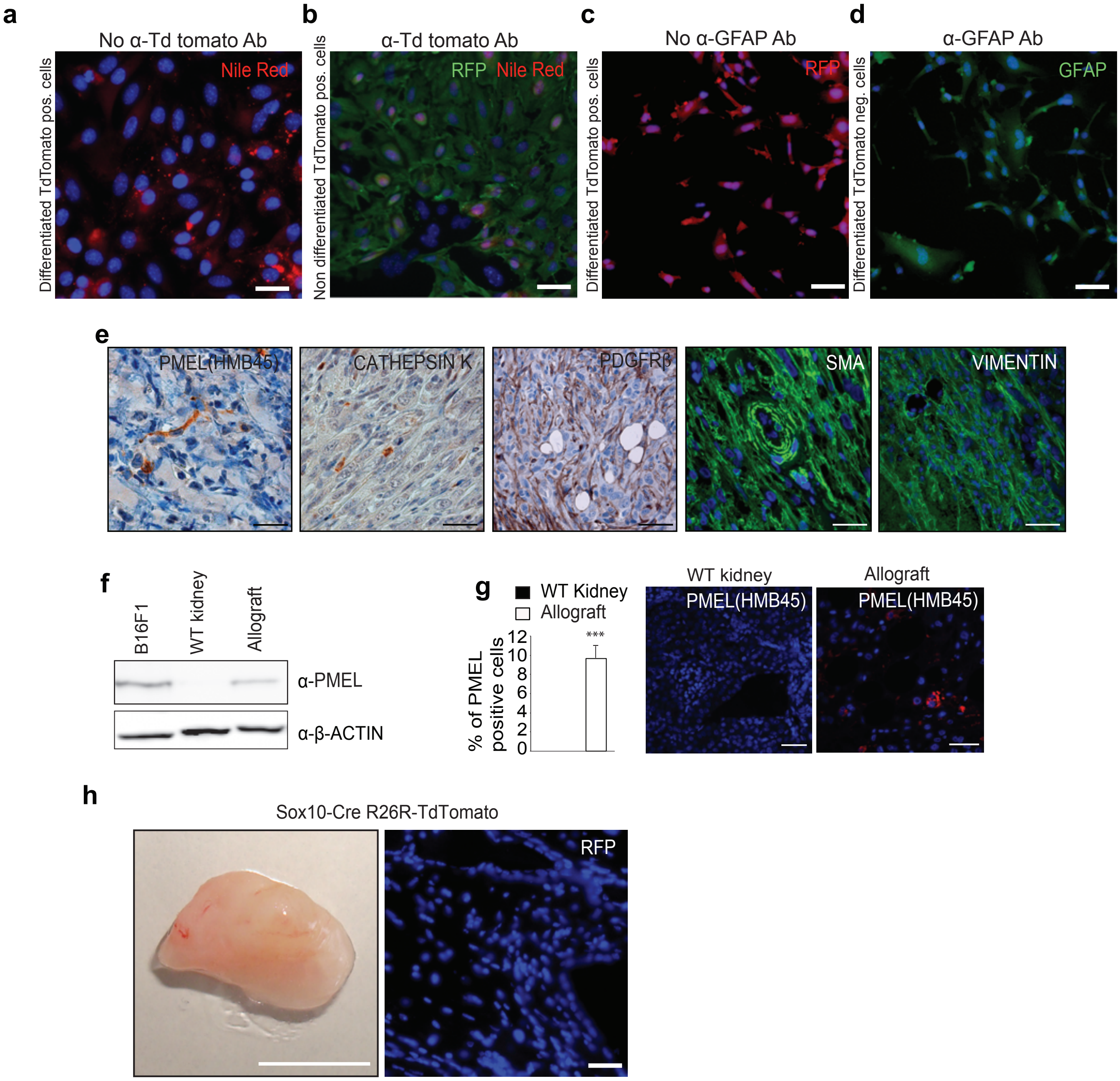

Supplementary Figure 10. Controls related to Figure 4.
(a) Negative control staining for RFP antibody in adipocyte differentiated TdTomato positive cells. Scale bar: 50 μm. **(b)** RFP immunostaining in non-differentiated tdTomato positive cells. Scale bar: 50 μm. **(c)** Negative control staining for GFAP antibody in neural crest differentiated tdTomato positive cells Scale bar: 50 μm **(d)** GFAP immunostaining in differentiated TdTomato negative cells. Scale bar: 50 μm. **(e)** Positive immunofluorescence or immunohistochemical staining of shRNA-*Tsc2+Cdkn2a* allograft derived from *Pax8-rtTA; TRE-LC1; R26R-tdTomato* mice for PMEL (HMB45), CATHEPSIN K, PDGFRβ, SMA and VIMENTIN. Scale bars: 50 μm. **(f)** Western blot analysis of PMEL in lysates from WT kidney, shRNA-*Tsc2+Cdkn2a* allograft derived from *Pax8-rtTA; TRE-LC1; R26R-tdTomato* mice and B16F1 mouse melanoma cell line. **(g)** Quantification of the percentage of PMEL positive cells in WT kidney and *Tsc2+Cdkn2a* allograft derived from *Pax8-rtTA; TRE-LC1; R26R-tdTomato* mice and immunofluorescence pictures of PMEL(HMB45) staining in the same samples. Graph depicts mean ± SD. Student's *t* test, n=3. *** P <0.001. Scale bars: 50 μm. **(h)** Image of shRNA-*Tsc2+Cdkn2a* tdTomato negative dissected AML allograft derived from *Sox10-Cre; R26R-tdTomato* kidney cells. Scale bars: 1 cm and 50 μm.

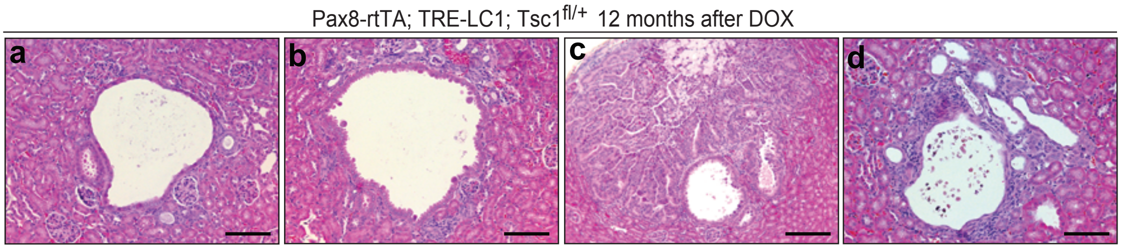

Supplementary Figure 11. Examples of lesions in aged *Pax8-rtTA; TRE-LC1; Tsc1$^{fl/+}$* mice
Representative histological images of kidneys 12 months after doxycycline treatment of 6-8 week old *Pax8-rtTA; TRE-LC1; Tsc1$^{fl/+}$* mice. **(a)** Simple cyst, **(b)** atypical cyst, **(c)** tumour with epithelial morphology and **(d)** lesion surrounding a cystic space and comprising closely packed cells with low cytoplasmic volume **(d)**. Scale bars: 150 µm.

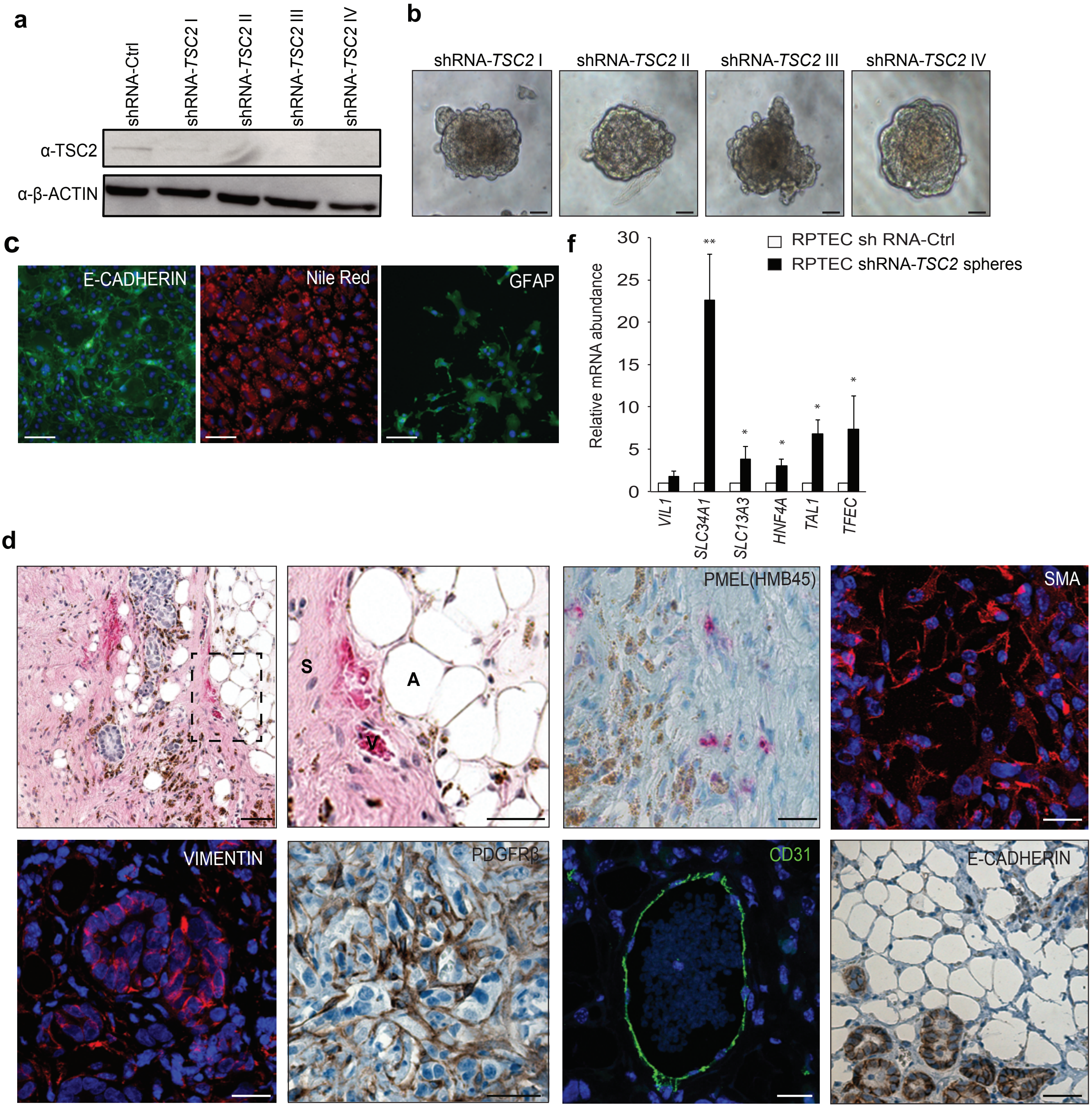

Supplementary Figure 12. Human renal AML-like model derived from immortalised RPTECs
(a) Western blot analysis of immortalised RPTECs transduced with four different lentiviruses expressing shRNA against *TSC2* (shRNA-*TSC2* I-IV). **(b)** Phase contrast images of spheres formed in suspension culture conditions from RPTECs infected with lentiviruses expressing shRNA-*TSC2* I-IV. Scale bars 100µm **(c)** Examples of differentiation of spheres into epithelial, adipocyte and glial lineages assessed with immunofluorescence stainings for E-CADHERIN and GFAP and Nile Red staining. Scale bars: 50µm. **(d)** mRNA expression analysis of indicated genes in RPTECs transduced with lentiviruses expressing an empty shRNA (shRNA-*Ctrl*) (n=3) or shRNA-TSC2 II and IV (n=3 experiments for each shRNA each, data pooled). Graph depicts mean ± SD. Student's *t* test, n=3. ** P <0.01; *** P <0.001. **(e)** Representative pictures of AML tumors derived from subcutaneous injections of *TSC2* knockdown RPTEC sphere cells into *SCID-beige* mice. Characteristic histological features of AML are indicated by letters, where A shows adipocytes, S shows spindle cells and V shows an aberrant blood vessel. Examples of positive immunofluorescence staining for HMB45, SMA, VIMENTIN, PDGFRβ, CD31 and E-CADHERIN. Scale bars: 50µm.

Figure 1a

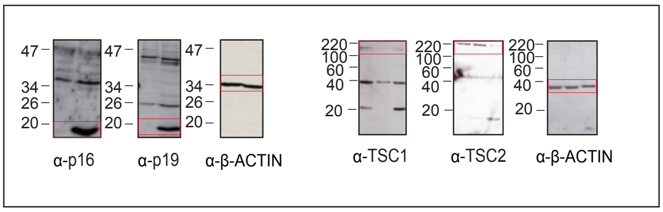

Figure 1b

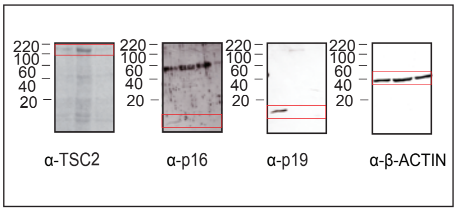

Supplementary Figure 1a

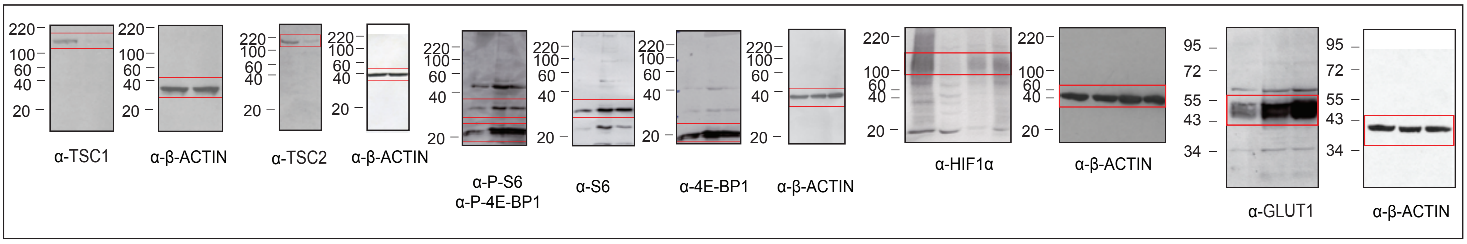

Supplementary Figure 1d

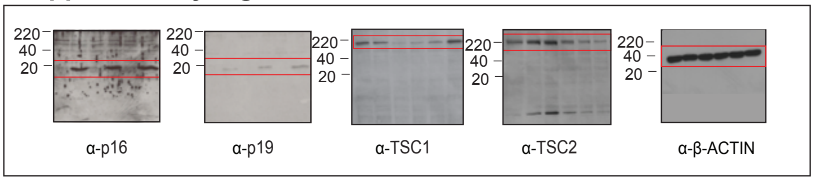

Supplementary Figure 2b

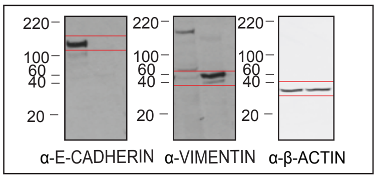

Suppl. Figure 3h

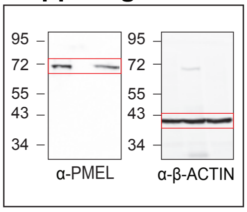

Supplementary Figure 3j

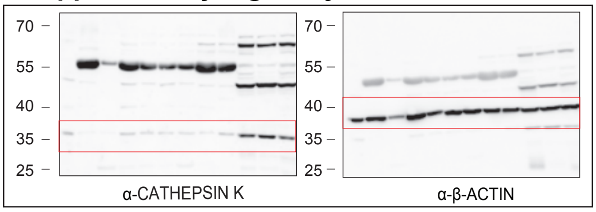

Supplementary Figure 6c

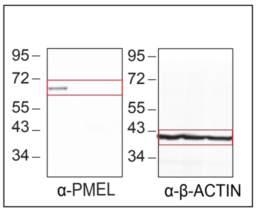

Supplementary Figure 7d

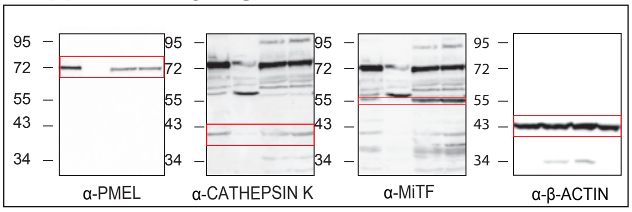

Supplementary Figure 9a

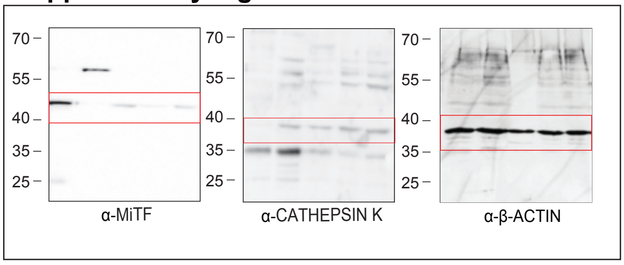

Supplementary Figure 10f

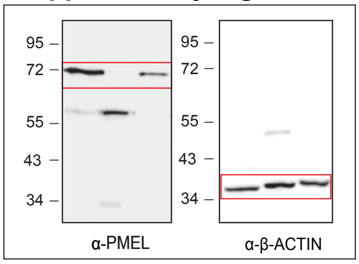

Supplementary Figure 12a

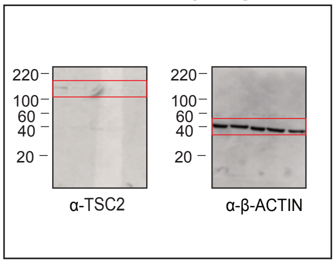

Supplementary Figure 13. Full scans of western blots presented in this publication.

Supplementary Table 1: List of primers used for real time PCR analyses

S12 mouse<br>F 5'-GAAGCTGCCAAAGCCTTAGA-3'<br>R 5'-AACTGCAACCAACCACCTTC-3	*Tal1 mouse*<br>F 5'-CACTAGGCAGTGGGTTCTTTG-3'<br>R 5'-GGTGTGAGGACCATCAGAAATCT-3'	*HNF4A human*<br>F 5'-CACGGGCAAACACTACGGT-3'<br>R 5'-TTGACCTTCGAGTGCTGATCC-3'
Lpl mouse<br>F 5'-GGGAGTTTGGCTCCAGAGTTT-3'<br>R 5'-TGTGTCTTCAGGGGTCCTTAG-3	*Hnf4a mouse*<br>F 5'-CACGCGGAGGTCAAGCTAC-3'<br>R 5'-CCCAGAGATGGGAGAGGTGAT-3'	*TAL1 human*<br>F 5'-CCAAAGTTGTGCGGCGTATC-3'<br>R 5'-CAGGCGGAGGATCTCATTCTT-3'
Sp7 mouse<br>F 5'-CATCTGCCTGACTCCTTGGGAC-3'<br>R 5'-GCTGAAAGGTCAGCGTATGGC-3'	*Tfec mouse*<br>F 5'-GGTCTCACGGATGCTCCTTG-3'<br>R 5'-TCCAGCGCATATCAGGATCATTA-3'	*TFEC human*<br>F 5'-GGACAACCACAACCTCATTGA-3'<br>R 5'-CACTTGATGTACTCCACTGATGC -3'
Bglap mouse<br>F 5'-CCTAGCAGACACCATGAG-3'<br>R 5'-TCTGATAGCTCGTCACAAG-3'	*Cubn mouse*<br>F 5'-CACTTTAGGTTGTGGTGGAACA-3'<br>R 5'-TTGCTGTCAAAGCTAATCTCCC-3'	*CUBN human*<br>F 5'-CTTGCAGCAGACTGTTGACAA -3'<br>R 5'-TGGCAGCTCAAGGGTGTTC -3'
Aqp1 mouse<br>F 5'- AGGCTTCAATTACCCACTGGA-3'<br>R 5'-GTGAGCACCGCTGATGTGA-3'	*Tubb3 mouse*<br>F 5'GCTCAGGGGCCTTTGGACATCTCTT-3'<br>R 5'-TGCAGGCAGTCACAATTCTC-3'	*VIL1 human*<br>F 5'-GGCAAGAGGAACGTGGTAGC -3'<br>R 5'-CGGTCCATTCCACTGGATGA-3'
Fmo1 mouse<br>F 5'-ACAGCCGACAGTATAAACATCCA-3'<br>R 5'-CCCTCCAGTAGTGCTGAGGAA-3'	*Kap mouse*<br>F 5'-GCTTTCCCCCTGTCAGAATTA -3'<br>R 5'-GTCCCTCTGTATGATCCCAGTT -3'	*FMO1 human*<br>F 5'-CCTGGCGGAAAAGGTGTTC -3'<br>R 5'-GGGTTGGGAGGGAATTTCTCA-3'
Vil1 mouse<br>F 5'-TCAAAGGCTCTCTCAACATCAC-3'<br>R 5'-AGCAGTCACCATCGAAGAAGC-3'	*Gfap mouse*<br>F 5'-CCCTGGCTCGTGTGGATTT-3'<br>R 5'-GACCGATACCACTCCTCTGTC-3'	*S12 human*<br>F 5'-TGCTGGAGGTGTAATGGACG -3'<br>R 5'-GGCGCTTGTCTAAGGCTTTG -3'
Slc34a1 mouse<br>F 5'-TGCCTCTGATGCTGGCTTTC-3'<br>R 5'-GATAGGATGGCATTGTCCTTGAA-3'	*Slc13a3 mouse*<br>F 5'-GGAAGGCCGATGCCTCTATG-3'<br>R 5'-GGAAGTTGGTGTCGAGGAAGT-3'	*SLC34A1 human*<br>F 5'-CCATCATCGTCAGCATGGTCT-3'<br>R 5'-GACAGCCAGTTAAAGCAGTCA-3'
Mlana mouse<br>F 5'-TCCCAGGAAGGGGCACAGACG-3'<br>R 5'-AGCGTTGGGAACCACGGGCT-3'	*Pmel mouse*<br>F 5'-TGACGGTGGACCCTGCCCAT-3'<br>R 5'-AGCTTTGCGTGGCCCGTAGC-3!	*SLC13A3 human*<br>F 5'-GGCGTGGCTATTGTCACCAT-3'<br>R 5'-CAGCCCCGATTCCTCACAG-3'
Ctsk mouse<br>F 5'-AAGTGGTTCAGAAGATGACGGGAC-3'<br>R 5'-TCTTCAGAGTCAATGCCTCCGTTC-3'	*Tyrp1 mouse*<br>F 5'-CCCTAGCCTATATCTCCCTTTT-3'<br>R 5'-TACCATCGTGGGGATAATGGC-3	
