# Supplementary material for: Evidence of renal angiomyolipoma neoplastic stem cells arising from renal epithelial cells
Source: Nat Commun. 2017 Nov 13;8:1466. doi: 10.1038/s41467-017-01514-3 (PMC5684212; doi:10.1038/s41467-017-01514-3)
Supplement: Supplementary file 3 — Description of Additional Supplementary Files [file 41467_2017_1514_MOESM3_ESM.pdf]

Description of Additional Supplementary Files

File Name: Supplementary Data 1
Description: RNA sequencing analysis of mRNA abundance in uninfected kidney cells (control), the total poulation (total) or sphere-selected population (spheres) of kidney cells after infection with lentivirus expressing shTsc2/shCdkn2a.

File Name: Supplementary Data 2
Description: Gene Set Enrichment Analysis: shTsc2/shCdkn2a total versus control, all genes >8 fold upregulated (154 identifiers converted to 126 Entrez genes).
